# Walnut extract protects against hepatic inflammation and toxicity induced by a high‐fat diet

**DOI:** 10.1002/fsn3.4405

**Published:** 2024-09-02

**Authors:** Gauhar Ali, Alam Zeb, Muhammad Usman, Salim Al‐Babili

**Affiliations:** ^1^ Department of Biotechnology University of Malakand Chakdara Pakistan; ^2^ Bioactive Lab, Centre of Excellence for Sustainable Food Security King Abdullah University of Science and Technology Thuwal Kingdom of Saudi Arabia; ^3^ Department of Biochemistry University of Malakand Chakdara Pakistan; ^4^ Department of Basic Sciences University of Veterinary and Animals Sciences Narowal Pakistan

**Keywords:** antioxidant, fatty liver, high‐fat diet, inflammation, phenolic compounds, walnut

## Abstract

A high‐fat diet (HFD) is one of the main causes of obesity and metabolic diseases. The liver is particularly affected by HFD causing metabolic dysfunction associated with fatty liver disease. Therefore, different strategies are used to mitigate the negative effects of HFD. This study aimed to assess the protective effects of walnut extract against HFD‐induced toxicity in mice. The mice were fed HFD and walnut extract alone or in combination. The walnut extract was analyzed for composition using high‐performance liquid chromatography with a diode array detector (HPLC‐DAD) and ultra‐high‐performance liquid chromatography with mass spectrometry (UHPLC–MS/MS). Serum lipid profile; liver histology; hepatic antioxidants such as catalase (CAT), superoxide dismutase (SOD), glutathione peroxidase (GSH‐Px), lipid peroxidation (TBARS), and reduced glutathione (GSH); inflammatory markers like IL‐6 and TNF‐α; and phospholipids were determined. Results showed that phenolic acids, epicatechin, catechin, benzaldehyde, and juglone were the main constituents in the extract. The HFD group showed increased hepatic fat accumulation as evidenced by biochemical and histopathological examinations compared to the control animals. The HFD group mice also showed increased body and cardiac weights, modified lipid profiles, decreased antioxidant status, and increased levels of hepatic inflammatory markers. The weights of the body and heart, lipid profiles, antioxidant contents (CAT, SOD, GSH‐Px, TBARS, and GSH), and pro‐inflammatory cytokines (IL‐6 and TNF‐α) were all normalized by consuming walnut extract. Similarly, the HFD group had significantly high amounts of hepatic lipase, phospholipid, and lysophospholipid levels, which were improved by walnut extract. In conclusion, walnut extract has been shown to play a unique role in promoting the recovery of liver damage caused by a high‐fat diet.

## Graphical abstract

The walnut extract contains gallic acid, caffeic acid hexoside, catechin, epicatechin, and benzaldehyde as the main components. Walnuts improved serum lipid profiles, and antioxidant, inflammatory markers, lipase, phospholipids, and lysophospholipids of the liver.

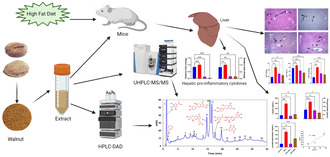

## 1 INTRODUCTION

With rising prevalence and incidence worldwide, obesity has become a significant health issue. Obesity is the result of consuming a high amount of dietary fat, which can lead to inflammation or disease. The use of high‐fat diets (HFD) is contributing to the increasing frequency of metabolic illnesses among humans. Unhealthy lifestyles and diets have led to a global increase in obesity caused by HFD. Overweight or obese individuals are at risk of developing diseases such as hyperlipidemia, type 2 diabetes, hypertension, and hypercholesterolemia (Furukawa et al., 2017). Heart diseases are mostly related to multiple factors, but one of the main causes is obesity and diet (Pan et al., 2014). Diabetes, one of the major metabolic diseases, has increased over the past few decades, and worldwide, more than 150 million people currently suffer from diabetes (Shaw et al., 2010). The causes of diabetes are reported to be high‐fat and high‐carbohydrate diets (Asmat et al., 2016).

HFD damages the energy balance in the body due to some comprehensive factors and lifestyle changes and causes obesity (Lizarbe et al., 2019). The WHO reports that nearly 2.5 million instances of diabetes caused by obesity pose a serious threat to public health. Previous studies showed that thermally oxidized dietary lipids produced liver toxicity, inflammation, and necrosis (Zeb & Akbar, 2018; Zeb & Mehmood, 2012). Oxidized lipids were the main driver of nonalcoholic fatty liver disease (Hoebinger et al., 2022), thus different amelioration strategies are warranted.

Walnut is one of those dietary supplements that has several health benefits. Walnut consumption has numerous known health advantages, including protection against diabetes and cardiovascular disease, and a decrease in both total and LDL cholesterol (Alasalvar et al., 2020). The walnut oil is primarily unsaturated, it has been linked to favorable effects on the lipid profiles. Walnuts are high in ω‐6 and ω‐3 polyunsaturated fatty acids, which are excellent dietary fatty acids, unlike most other nuts, which primarily include monounsaturated fatty acids and the least amount of polyunsaturated fatty acids. It is suggested by clinical studies that ω‐3 polyunsaturated fatty acids might be crucial for minimizing the risk of coronary heart disease (Sala‐Vila et al., 2022). Several mechanisms were suggested for that action, including antithrombotic, antiarrhythmic, and hypolipidemic roles (Bae et al., 2023; Naghshi et al., 2021).

Previous studies indicated that the early phases of non‐alcoholic fatty liver disease were caused by imbalanced production of proinflammatory adipokines produced from fat, such as tumor necrosis factors (Kanda et al., 2006; Lê et al., 2011). Studies have also shown that the main infective role of adipocyte apoptosis by macrophages in adipose tissues is to induce inflammation and metabolic disruption, resulting in hepatic lipid accumulation (Cinti et al., 2005). Additionally, a recent study discovered that supplementing with walnuts improved lipid‐induced hepatic steatosis in rats by modifying lipoprotein synthesis and hepatic fatty acid influx (Mateș et al., 2022). In high‐fat diet‐induced C57BL/6 mice, walnuts are protected against cognitive damage by controlling the dysfunction of the synaptic and mitochondrial system through the c‐Jun N‐terminal kinase signaling and apoptosis pathway (Moon et al., 2022). Our recent study showed that walnut extract improved the side effects of lipids oxidized thermally (Ali & Zeb, 2024). However, the effects of an unoxidized HFD rich in animal tallow still need to be explored. A recent review and meta‐analysis of randomized controlled trials showed that walnuts can be beneficial for blood lipids. However, there is a paucity of research on the effectiveness of walnuts in the therapy of high‐fat diet‐induced liver inflammation and damage. This study was, therefore, aimed to see how effective walnut extract was against HFD‐induced toxicity in mice.

## 2 MATERIALS AND METHODS

### 2.1 Materials

Methanol, eugenol, catechin, quercetin, and acetic acid were from Sigma‐Aldrich (Hamburg, Germany). Commercially available kits (Merck Pvt Ltd, Karachi, Pakistan) of total cholesterol (TC), total triglycerides (TG), low‐density lipoprotein cholesterol (LDL‐c), high‐density lipoprotein cholesterol (HDL‐c), glutathione peroxidase (GSH‐Px), and alanine aminotransferase (ALT) were used. Kits such as catalase, superoxide dismutase, glutathione peroxidase, lipase, and phospholipids were from Cusabio Technology LLC (Houston, USA), HiPure Total RNA kit was from Magen Biotechnology Co, Ltd, Guangzhou, China, whereas cDNA kit was from Zokeyo, Wuhan, China. All chemicals and reagents comprised of high purity and analytical grade certified by the ACS.

### 2.2 Sample preparation

Walnut (*Juglans regia*) samples were purchased from the local market in Mingora, Swat. The walnut kernels were separated from the shells and husk and were ground and stored at −20°C. The walnut extract was prepared by mixing 1 kg of walnuts in 2 l of methanol (100%). The mixture was continuously shaken at a temperature of 25°C for 48 h. The mixture was filtered using Whatman filter paper (Sigma‐Aldrich, Karachi, Pakistan) and the extract was evaporated using vacuum distillation at temperature (−4°C) to obtain a dry extract.

### 2.3 HPLC‐DAD analysis

An Agilent 1260 Infinite System in reverse phase was utilized to identify the phenolic compounds present in walnut extract. A C18 high‐resolution column (Agilent Technologies, Waldbronn, Germany) with a specification of 4.6 × 100 mm was used, which was controlled at a temperature of 25°C. In the binary solvent system, solvent A (methanol–acetic acid–water, 10: 2: 88, v/v/v) and solvent B (methanol–acetic acid–water, 90: 2: 8, v/v/v) were used. The flow rate (1 mL/min), injection volume (50 μL), and elution program were as per the actual method (Zeb, 2015). The spectra were captured between 200 and 700 nm, and chromatograms were obtained at 320 nm using Chemstation software (Agilent Technologies, Germany). The standards used were gallic acid, benzaldehyde, cinnamic acid, eugenol, catechin, coumarin, quercetin, and ellagic acid, and quantified mg/g of DW of the extract.

### 2.4 UHPLC–MS/MS analysis

To confirm the identified compounds, the walnut extract was investigated using UHPLC–MS/MS. Agilent Zorbax Eclipse XDB C18 (Agilent Technologies, Waldbronn, Germany) column and UHPLC UltiMate‐3000 (Thermo Scientific, Bremen, Germany) System were used for the separation (Ali & Zeb, 2024). The major peaks were targets of the MS analysis. The Q‐Executive Plus Orbitrap MS (Thermo Scientific, Bremen, Germany) was preequilibrated in positive mode. The scan ranges from 100 to 1500 m/z.

### 2.5 Analysis of tallow

The samples of tallow in HFD were examined using GC–MS (model 5977B, Agilent Technologies, Santa Clara, USA). The experimental setup included a 36‐min runtime, an oven temperature range of 70–270°C, a 2‐min equilibration period, a 1‐microliter injection volume, and a 24‐milliliter per minute helium gas flow. The MS parameters consisted of a mass range of 30–650 and a temperature of 250°C. Compounds were quantified using the percent peak area as reported previously (Zeb & Ullah, 2015).

### 2.6 Animal study

The Swiss Albino mice (male, aged 6–8 weeks, weighing 30–35 g) were acquired from the Veterinary Research Institute, Peshawar. All studies involving animal care and experimentation were approved by the ethics committee of the Department of Biotechnology, following Helsinki standards. The research was subsequently accepted by the Graduate Studies Committee of the Department and the Advanced Studies and Research Board of the University of Malakand (No. UOM/Admin/2022/955). The mice were kept in a climate‐controlled space with a 12‐h light and dark cycle, temperature (24°C), and humidity (45%). They were provided with regular water and food in their cages. The mice were acclimatized for 7–10 days prior to feeding. The mice were randomly divided into four groups: (1) control; (2) mice fed with HFD of 2.8 mg/g body weight (BW); (3) mice fed with walnut extract (WE) at a dose of 3.3 mg/kg BW with HFD; and (4) mice fed with WE at a dose of 6.6 mg/kg, BW with HFD. They were assigned as control, HFD, HFD + WE1, and HFD + WE2 labels (Ali & Zeb, 2024). Each group consisted of five replicate animals. The animals were orally fed for a duration of 4 weeks (Fettach et al., 2024) and their body weight was measured weekly. The HFD consisted of tallow 2.85 g/kg.

After completing the feeding cycle, the animals were sacrificed and samples of blood were processed in specific gel tubes to obtain serum using centrifugation, which was then stored at −20°C. The liver was removed and then stored in a 10% formalin solution.

### 2.7 Body and organs weight

Body, liver, and heart weights were measured using an analytical balance. The liver and heart were removed from all groups, weighed accordingly, and then stored in formalin (10%).

### 2.8 Biochemical analyses

Using a biochemistry analyzer (model BA‐88A, Mindray Biomedical Electronics Co., Ltd, Shenzhen, China), the serum lipid profiles including TC, triglycerides, HDL‐c, and LDL‐c were determined. The CRP levels were also quantitatively measured in the serum. Additionally, the levels of AST and ALT were measured following the manufacturer's protocols.

### 2.9 Histological examination

Three mice were chosen at random from each group to receive liver samples, which were then fixed in 10% (v/v) paraformaldehyde/PBS. The slides were prepared, and histological observations were performed through a procedure reported recently in our work (Ali & Zeb, 2024).

### 2.10 Total glutathione contents

The liver samples were homogenized using handheld laboratory tissue grinder in the presence of buffer and ice. Total glutathione contents (reduced) were extracted from the homogenized sample (100 mg), and mixed with 2 mL of buffer (pH 7.5) and phosphoric acid (8 mL of 3%). After 30 min of shaking, 0.5 μL of the sample was mixed with a buffer solution of 1500 μL. After that, dithio‐nitrobenzene was added and incubated for 2 min (37°C). A UV–visible spectrophotometer (Shimadzu, Tokyo, Japan) was utilized. At 412 nm, absorbance was measured in comparison to a blank reference. The resulting value was given in mol/g (Zeb & Akbar, 2018).

### 2.11 Catalase

The catalase in the liver samples was measured using the quantitative enzyme immunoassay method as per the manufacturer protocol (Cusabio Technology LLC, USA). Standards and samples (100 μL each) were reacted with precoated CAT antibodies followed by peroxidase reaction, substrate addition, and incubation. The reaction was stopped, and the intensity of color was determined at 450 nm. The values of CAT were expressed as pg/mL. Catalase (CAT) was analyzed by PHC Diagnostics, Lahore, Pakistan.

### 2.12 Lipid peroxidation

According to the previously published protocol, lipid peroxidation in the liver was assessed as TBARS (Zeb & Ullah, 2016). The TBA reaction with MDA or TBARS resulted in a purple color, and a spectrophotometer was used to determine the absorbance of this color at 532 nm. The TBARS values were calculated from the standard calibration curve of MDA and were expressed as μmol/g.

### 2.13 Superoxide dismutase

The ability of the liver homogenates to scavenge free radicals was assessed using superoxide dismutase (SOD) using a quantitative enzyme immunoassay method as described recently (Ali & Zeb, 2024). The SOD was analyzed by PHC Diagnostics, Lahore, Pakistan. Microplates with an antibody were mixed with standards and samples of 100 μL each. Treatment with peroxidase was followed by incubation for 30 min before adding a stop solution. The standard curve was used to calculate SOD (pg/mL).

### 2.14 Glutathione peroxidase

Glutathione peroxidase assay of the liver samples was also based on enzyme immunoassay protocol. Glutathione peroxidase (GSH‐Px) was analyzed by PHC Diagnostics, Lahore, Pakistan. Microplates were precoated with a specific GSH‐Px antibody mixed with 100 μL each of standards and samples. This peroxidase treatment was followed by a substrate addition and incubation, then reaction stoppage. The GSH‐Px (pg/mL) was estimated using the standard curve.

### 2.15 Hepatic inflammatory markers

The total RNA was extracted from liver samples using an RNA kit as per the method described recently (Ali & Zeb, 2024). The real‐time expression of the targeted genes (IL‐6 and TNF‐alpha) was carried out using cDNA (template), and the primers used are shown in the Supplementary File, Table S1. The relevant Ct values of the samples were evaluated in comparison to controls and control samples with reference to housekeeping genes. Hepatic inflammatory markers were studied by PHC Diagnostics, Lahore, Pakistan.

To carry out the RT‐qPCR procedure, an improved method was used. With the mixture having a total reaction volume of 15 μL, the qRT‐PCR was performed. The mixture contained cDNA (1 μL), SYBR green mix (10 μL), and primers (0.5 μM each). The relevant Ct values of the samples were measured with reference to housekeeping genes (GAPDH). The amplification conditions are 40 cycles of 95°C for 5 s and 60°C for 20 s, followed by 95°C for 30 s (Darabi et al., 2020).

### 2.16 Lipase and phospholipids in the liver

The lipase and phospholipids in the liver were measured and expressed as ng/ml using the enzyme immunoassay technique as per the manufacturer's instructions. PHC Diagnostics, Lahore, Pakistan, had determined phospholipids and hepatic lipase contents.

### 2.17 Statistical analysis

A one‐way ANOVA with Dunn's multiple‐comparisons test was utilized to compare the data using GraphPad Prism (version 10.2.1; Graph Pad Software, USA). Two‐way ANOVA with Tukey's multiple‐comparisons test and an unpaired *t*‐test was employed for gene expression analysis. At various p values, differences across groups were deemed significant.

## 3 RESULTS

### 3.1 Analysis of composition

**FIGURE 1:**
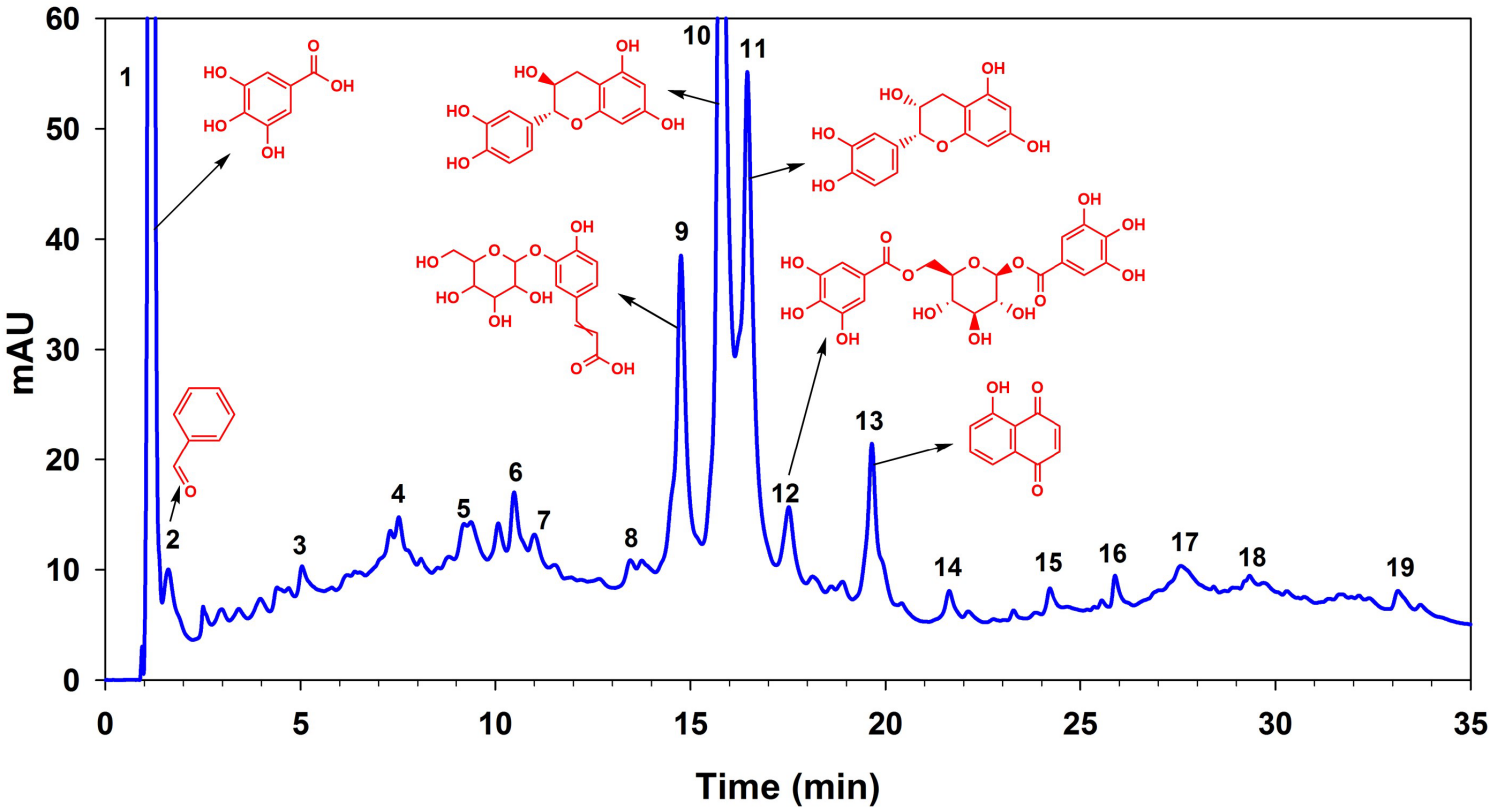
HPLC‐DAD chromatogram of the walnut extract at 320 nm. Some of the major compounds with structures are shown.

Figure 1 shows a chromatogram of the HPLC‐DAD separation, identification, and structures of phenolic compounds present in the extract of walnuts prepared in methanol. Nineteen phenolic compounds were identified in the walnut extract with identification characteristics, that is, *λ*max, *m/z*, reference ion, and MS2 as shown in Table 1. Of the extract's characterization, 75.38% could be explained by the 753.8 mg/g of identified and measured components. Gallic acid was the first compound eluted at 1.1 min having the highest quantity among all compounds. Benzaldehyde was the second eluted compound with composition of 45.8 mg/g. Subsequently, cis‐cinnamic acid quantity of 16.7 mg/g was present. The fourth compound eluted was 2,4‐dimethylbenzaldehyde having a concentration of 16.2 mg/g. Peak 5 was annotated as *p*‐hydroxybenzalacetone with concentration of 31.7 mg/g. Peak 6 was isoferulic acid with a concentration of 26.3 mg/g. Peaks 7 and 8 were eugenol and methyl jasmonate having concentrations less than 15 mg/g. Caffeic acid hexoside was present as peak 9 was ranked second in terms of concentration (120.8 mg/g). Catechin and epicatechin were peaks 10 and 11 having concentrations of 93.5 and 66.5 mg/g, respectively. Peak 12 was digalloyl‐glucoside and peak 13 was juglone with a concentration of 17.9 mg/g. Peak 14 was coumarin (3.47 mg/g). Peaks 15–17 were flavonoids, that is, 8‐hydroxy‐quercetin, quercetin, and hesperidin, respectively, with relatively similar amounts. Naringenin chalcone (3.01 mg/g) was at peak 18 having *λ*max of 282 nm and *m/z* of 273.0756. The last compound was ellagic acid with a concentration of 3.17 mg/g. These results indicated that gallic acid (25.95%), caffeic acid hexoside (12.08%), catechin (9.35%), epicatechin (6.65%), and benzaldehyde (4.58%) were the top 5 phenolic compounds in decreasing order of quantity above than 50 mg/g. These five compounds were contributing to 58.61% of the total quantity of the extract.

**TABLE 1:** Metabolite composition of walnut extract (mg/g) using HPLC‐DAD and UHPLC–MS/MS.

Peak No	Rt (min)	Identity	*λ*max (nm)	*m/z*	Ref. ion	MS2	Quantity^a^ (mg/g)
1	1.1	Gallic acid	271	170.9991	[M + H] + 1	152.0705	259.5 ± 4.1
2	1.6	Benzaldehyde	330	107.0495	[M + H] + 1	84.0449	45.8 ± 1.4
3	5.1	*E*‐Cinnamic acid	278	166.0867	[M + NH4] + 1	149.0579, 131.0492	16.7 ± 0.3
4	7.4	2,4‐Dimethylbenzaldehyde	209	135.0805	[M + H] + 1	121.0625, 119.09	16.2 ± 0.2
5	9.2	p‐Hydroxybenzalacetone	321	145.0649	[M + H‐H2O] + 1	98.9756	31.7 ± 0.7
6	10.4	Isoferulic acid	258	195.0621	[M + H] + 1	165.0534	26.3 ± 0.7
7	10.9	Eugenol	281	165.0911	[M + H] + 1	145.0502	14.3 ± 0.2
8	13.5	Methyl Jasmonate	206	225.1484	[M + H] + 1	132.9558, 98.9757	13.5 ± 0.1
9	14.7	Caffeic acid hexoside	328	343.1214	[M + H] + 1	181.0524, 165.0867	120.8 ± 1.7
10	15.8	Catechin	280	291.0896	[M + H] + 1	165.0546, 139.0385, 123.0433	93.5 ± 1.2
11	16.5	Epicatechin	280	291.0860	[M + H] + 1	165.0546, 139.0389, 123.0441	66.5 ± 0.4
12	17.4	1,6‐Digalloyl‐glucose	363, 264	484.5856	[M + H] + 1	360.0735, 331.0742, 155.0325	12.4 ± 0.2
13	19.6	Juglone	407, 234	175.0412	[M + H] + 1	158.0423	17.9 ± 0.3
14	21.6	Coumarin	272	147.0502	[M + H] + 1	131.1532, 91.0521	3.47 ± 0.1
15	24.2	8‐Hydroxy‐quercetin	388, 262	335.0395	[M + H] + 1	303.0132, 293.9610	2.84 ± 0.03
16	25.9	Quercetin	370, 260	303.0498	[M + H] + 1	257.0445, 229.0494,165.0181	2.84 ± 0.03
17	27.6	Hesperidin	323, 270	611.2103	[M + H] + 1	488.1512, 325.1101, 287.0921	3.25 ± 0.1
18	29.3	Naringenin chalcone	282	273.0756	[M + H] + 1	153.1814, 98.9712	3.01 ± 0.03
19	33.1	Ellagic acid	367, 275	303.0133	[M + H] + 1	285.0027, 275.0118	3.17 ± 0.03
Total							753.8

^a^ Values are mean with standard deviation (*n* = 3).

The GC–MS analysis revealed 16 constituents (Supplementary File, Figure S1 and Table S2): ethylbenzene, tetradecanoic acid, pentadecanoic acid, E‐9‐hexadecenoic acid, n‐hexadecanoic acid (13.447%), heptadecanoic acid, (E)‐9‐octadecenoic acid, oleic acid (26.599%), NAE 15:2 (7.633%), octadecanoic acid (13.238%), (Z, Z)‐9,12‐octadecadienoic acid, tetracosane, bis(2‐ethylhexyl) phthalate (29.41%), eicosane, (Z)‐9‐octadecenoic acid, and cholesterol.

### 3.2 Body and organ weight

**FIGURE 2:**
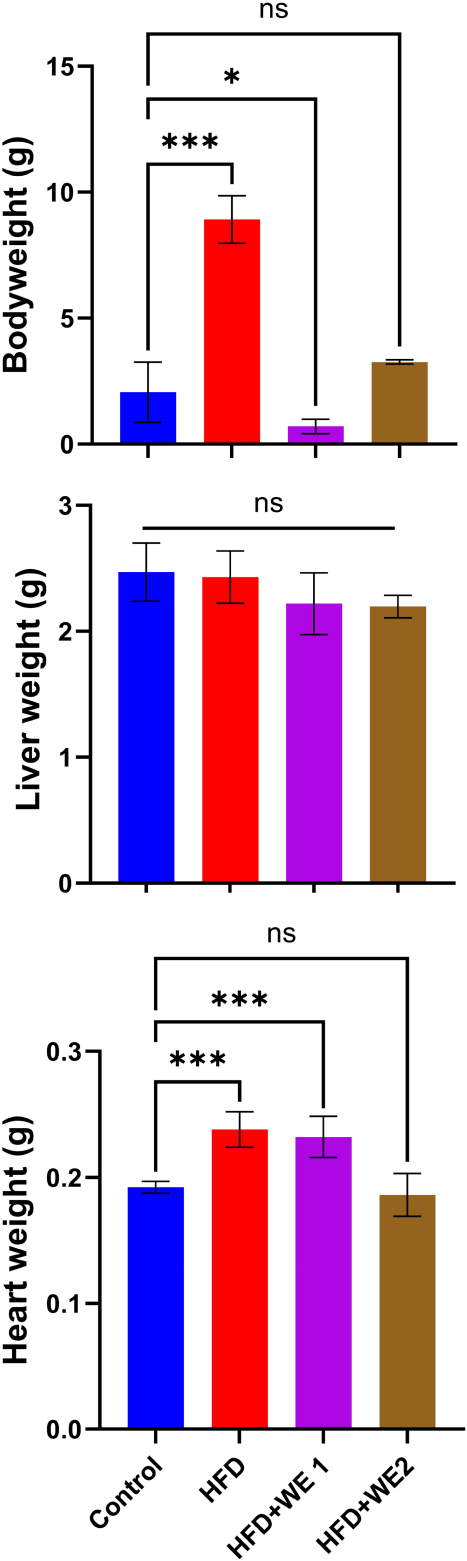
Weight effects of walnut extract on the body, liver, and heart. Dunnett's multiple‐comparison tests were used to show the data as mean with a standard deviation of replicates (*n* = 5), ns = no significant, *p = .033, ***p < .001 against control in each treatment.

Figure 2 illustrates a significant increase in body weight in the HFD groups compared to the control and treatment groups. However, once WE1 was supplemented, the mice's body weight decreased significantly. Interestingly, when comparing the body weight of HFD + WE2 group to the control, there was no noticeable change. The liver weight remained unchanged across all treatment groups, including the control group. On the other hand, there was a significant increase in heart weight in the HFD and HFD + WE1 compared to the control. The heart weight of all groups was similar to that of the control group. These results suggest that body and heart weight was normalized by supplementation of walnut extracts.

### 3.3 Serum lipid profile

**FIGURE 3:**
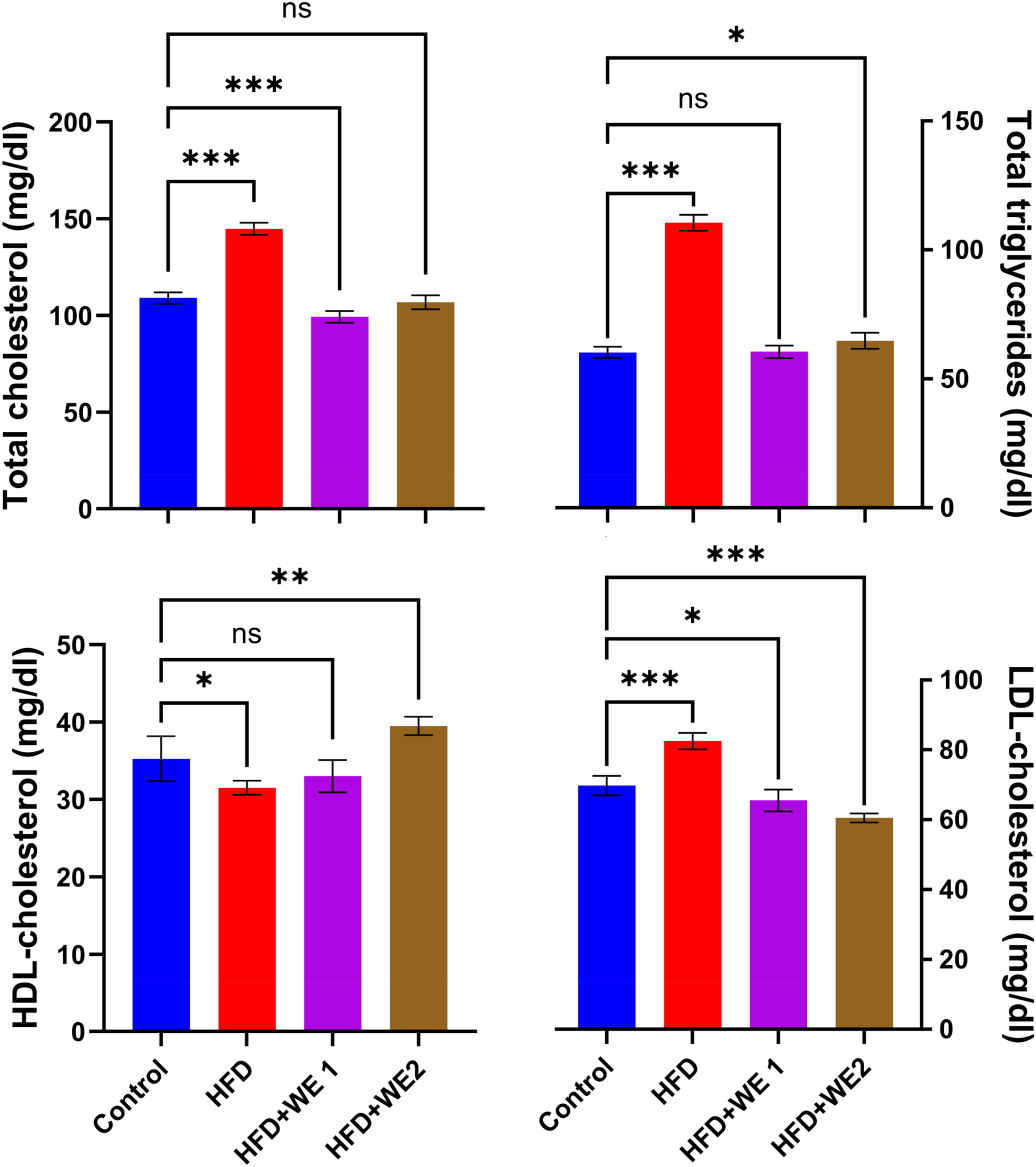
The impact of walnut extract on the blood lipid profile of mice, including total cholesterol, total triglycerides, low‐density lipoprotein cholesterol, and high‐density lipoprotein cholesterol. Data presented as mean with standard deviation of replicates (*n* = 5), utilizing Dunnett's multiple‐comparison tests with ns = no significant, *p = .033, **p = .002, and ***p < .001 against control in each treatment.

Mice on an HFD had significantly elevated serum levels of TC and TG. The levels of TC and TG decreased substantially in the HFD + WE1 and HFD + WE2 groups compared to the control group (Figure 3). The HFD group had the lowest serum levels of HDL, while the HFD + WE2 group had the highest levels, which were significantly different from the HFD group. The LDL‐c levels of all three groups were statistically different from the control group, showing a decrease when walnut extracts were used. These findings suggest that walnut extracts significantly mitigated the effects of HFD on the serum lipid profile.

### 3.4 Liver histology

**FIGURE 4:**
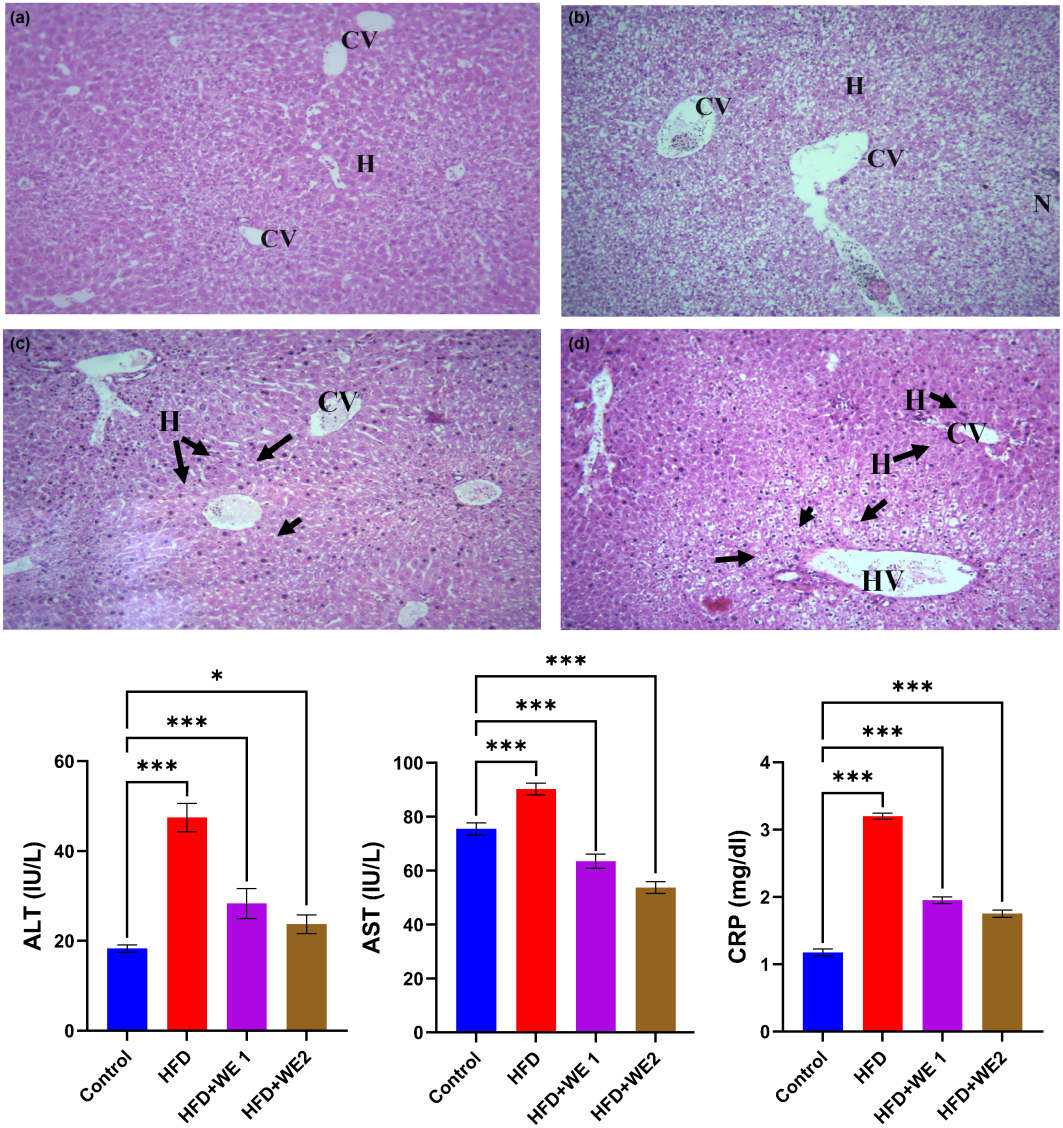
Impact of walnut extract on mouse liver function tests and liver histology. (a) Control, (b) HFD, (c) HFD + WE1, and (d) HFD + WE2. The following terms are acronyms: C‐reactive protein (CRP), aspartate aminotransferase (AST), hepatocyte (H), sinusoidal cord (small arrow), central vein (CV), necrosis (N), and C‐reactive protein (CRP). The data are shown as the mean of five replicates with a standard deviation. Dunnett's multiple‐comparison tests reveal that in each treatment, *p = .033 and ***p < .001 compared to the control group.

Histological findings reveal normal liver histology for control (Figure 4a), and liver damage in mice fed with HFD (Figure 4b). Strong indicators of liver damage include elevated ALT, AST, and CRP levels, which were observed in the HFD group. As shown in Figure 4c,d, the hepatocyte patterns of the walnut‐supplemented groups appear normal in comparison to the HFD group. Furthermore, histological observations indicated that lipids storage occurred in the liver in the HFD group was higher than in the walnut extracts supplemented group.

### 3.5 Hepatic antioxidant status

**FIGURE 5:**
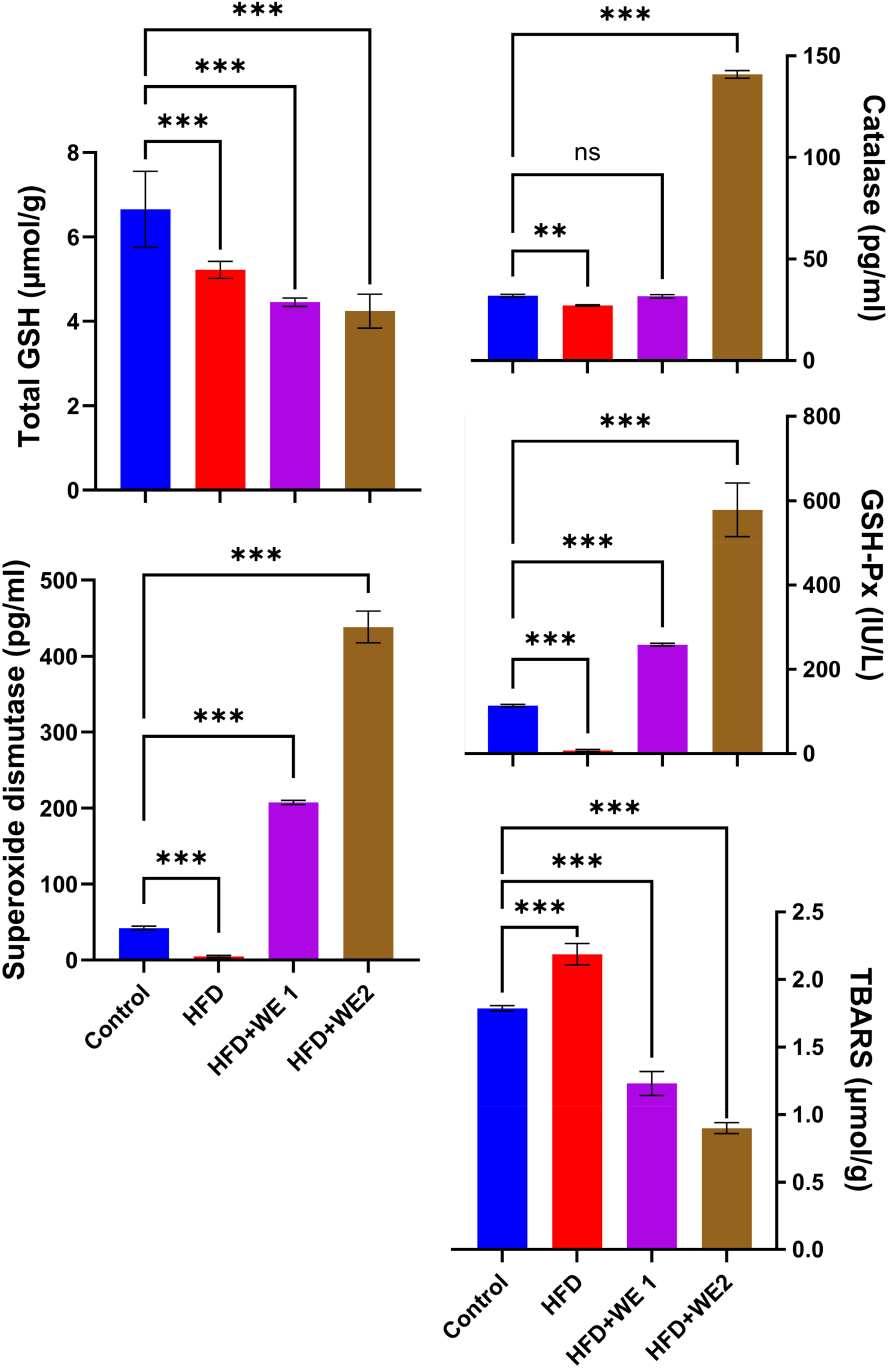
Influence of walnut extract on mice's liver antioxidant status (catalase, total reduced glutathione, glutathione peroxidase, superoxide dismutase, and thiobarbituric acid reactive substance). The data are shown as the mean of five replicates with a standard deviation. Using Dunnett's multiple‐comparison tests, the results for each therapy were **p = .002, and ***p < .001 versus control.

As demonstrated in Figure 5, the GSH levels declined significantly in all treatment groups in comparison to the control group. When compared to HFD and control groups, the HFD mice had significantly lower hepatic CAT levels. The HFD + WE2 group had the highest CAT (140.98 pg/mL) as compared to control. Similarly, the livers of the HFD group had significantly lower SOD levels than the control group. When compared to the HFD and control groups, the treatment group that received walnut extracts (HFD + WE1 and HFD + WE2) showed a considerable increase in SOD levels. The addition of walnut extracts to the HFD significantly increased glutathione peroxidase activity. Compared to the control group (113.8 IU/L), the HFD + WE2 group had fivefold higher levels (578.4 IU/L). In comparison to the control group, the HFD group had noticeably higher TBARS levels. The addition of walnut extracts resulted in a dose‐dependent reduction in TBARS levels, with 1.23 and 0.899 μmol/g for the last two treated groups. These findings suggest that walnut extracts have a significant positive impact on the liver's antioxidant status.

### 3.6 Hepatic inflammation

**FIGURE 6:**
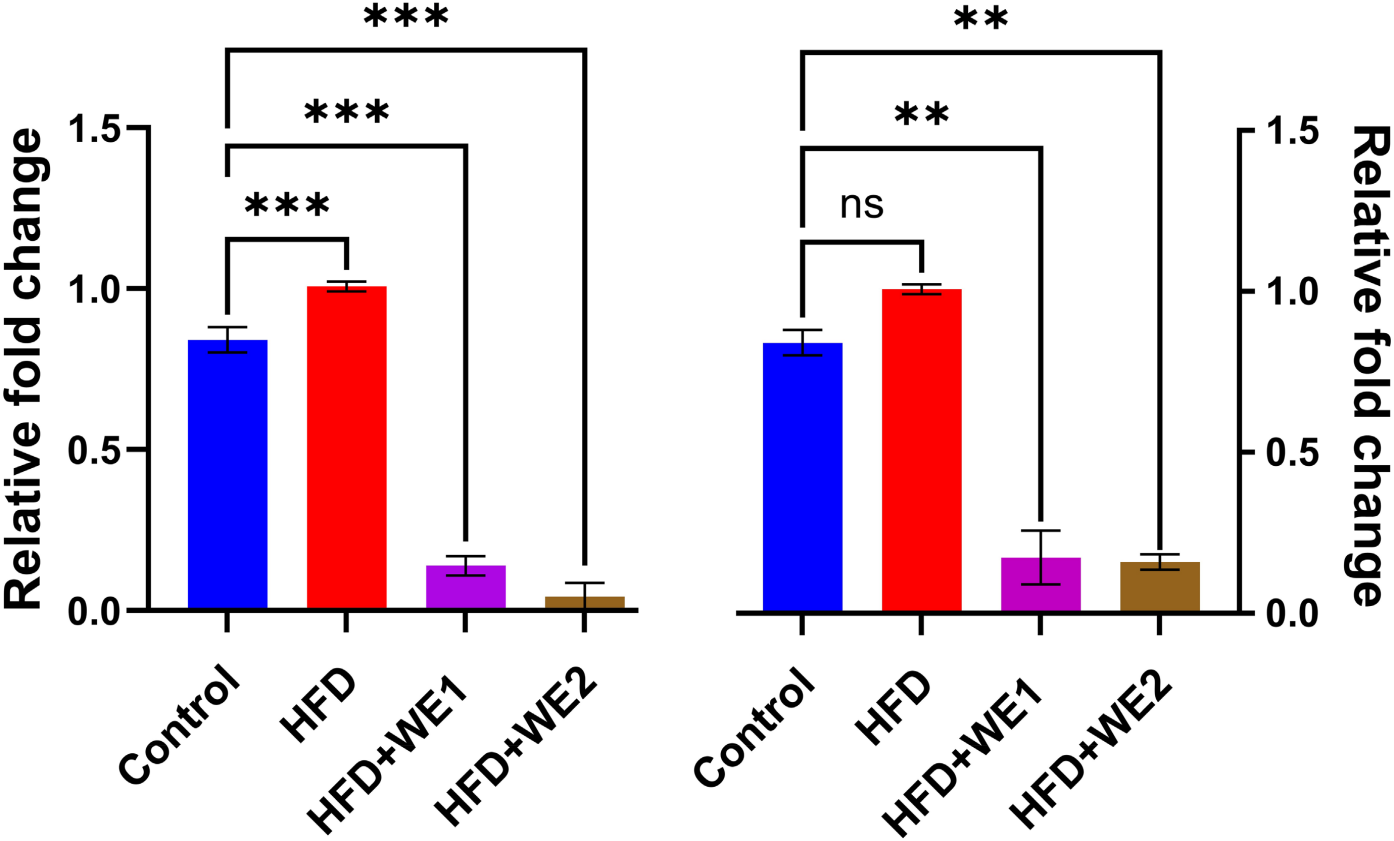
Impact of walnut extract on the relative fold change in inflammatory marker expression in mouse liver groups. Using Dunnett's multiple‐comparison tests, the data were shown as mean with a standard deviation of replicates (*n* = 3), ns, no significant, **p = .002, and ***p < .001 against control in each treatment.

The results demonstrated in Figure 6 showed that, in contrast to the HFD + WE group, the levels of selected genes were elevated in the HFD group. When comparing the HFD‐fed mice to the control group, the level of IL‐6 was considerably greater (*p* > .05). IL‐6 expression was significantly downregulated in the HFD + WE‐treated groups. Comparing the HFD + WE group to the control group, there was a notable upregulation of TNF‐α expression and a downregulation of it. These findings demonstrated that walnut extracts shielded the liver from HFD‐induced inflammation.

### 3.7 Hepatic lipase and phospholipids

**FIGURE 7:**
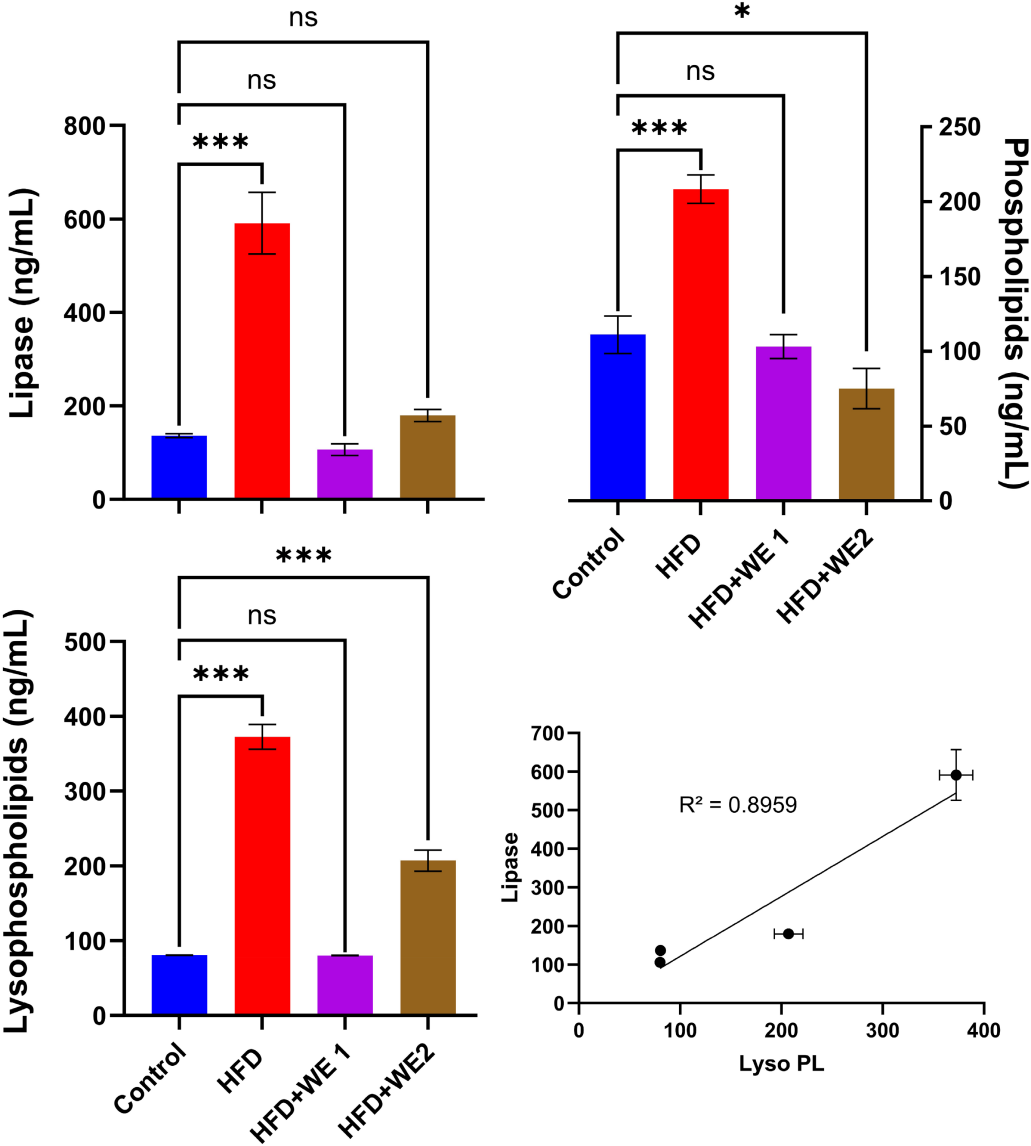
Impact of walnut extract on liver phospholipid content and lipase levels, as well as lipase and lysophospholipid content relationships. The data are shown as mean with replicates' standard deviation (*n* = 5). Using Dunnett's multiple‐comparison tests, each treatment's *p = .033 and ***p < .001 against control were found in each case.

Figure **7** demonstrated that hepatic lipase levels (591.1 ng/mL) significantly elevated after HFD supplementation than the control group (136.2 ng/mL). With no apparent distinction from the control group, the lipase concentrations were considerably lowered by the addition of walnut extract. The amount of hepatic phospholipids contents was also significantly elevated in the group fed with HFD (208.3 ng/mL) as compared to the control (110.9 ng/mL). The administration of walnut extracts significantly declines the amounts of phospholipids in the liver. The amount of lysophospholipids was significantly enhanced with HFD and reduced by walnut extracts. On plotting the lipase contents against the lysophospholipids, a strong linear correlation (*R* ^2^ = .8959) was observed as shown in Figure 7. These findings confirmed that HFD alters the lipid metabolism in the liver and walnut extracts ameliorated its effects.

## 4 DISCUSSION

The leading cause of obesity is the consumption of high‐fat diets. Obesity is directly related to serious health complications, such as diabetes, high blood pressure, high cholesterol levels, and related heart conditions, and is primarily caused by the consumption of HFD. According to the World Health Organization (WHO), at least one of eight people worldwide is obese, making it a global problem affecting a billion individuals. Trimming the fat has become a worldwide concern.

Research indicates that obesity, which can lead to DNA damage and other health issues, is largely caused by HFD (Setayesh et al., 2019). Consequently, researchers from around the world are now investigating the molecular causes of obesity and how HFD may contribute to it. Animal tallow has been found to be rich in fatty acids including oleic acid, octadecanoic acid, and bis(2‐ethylhexyl) phthalate (DEHP). An epidemiological study has shown a strong correlation between several phthalate metabolites and abdominal obesity in adult males (James‐Todd et al., 2016). Evidence suggests that DEHP interferes with the metabolism of fatty acids in adipocytes and adipose tissue (Klöting et al., 2015). Therefore, it can be concluded that the presence of DEHP in the HFD may be one of the reasons causing NAFLD. In light of the negative consequences of synthetic substances, natural resources and ingestible functional foods such as walnuts are becoming increasingly important (Atanasov et al., 2015).

The extract of walnut kernel contains 19 compounds, which consist of phenolic acids or their derivatives, coumarins, juglone, catechins, and flavonoids. A detailed profiling using untargeted metabolomic approach of the different walnuts from Pakistan revealed 135 metabolites, categorized into 14 classes: hydrocarbons, phenolic acids, ketones, flavonoids, lipids, carboxylic acids and amides, phenyl alcohols and aldehydes, oxygenated hydrocarbons, glycosides, phenolic acids, hydrocarbons, sugar alcohols and acids, and vitamins (Zeb et al., 2024). Phenolic acids were the main components in samples from Portugal (Pereira et al., 2007), however, none of these were identified in the present study. In black walnut kernels, 16 phenolics were reported which include phenolic acids, flavonoids, and catechins, with ellagic acid as a predominate compound (Vu et al., 2018). Comparably, juglone and flavonoids were found in Chinese samples along with ellagic acid, gallic acid, ferulic acid, sinapic acid, and caffeic acid (Wu et al., 2021). These findings showed that the phenolic chemicals found in the Pakistani walnut samples were much higher than those found in reported walnuts contributing to the beneficial effects against HFD.

It was found that walnut supplements effectively reduce irregular weight gain by decreasing body fat. These supplements also help regulate lipid profiles including TG, TC, HDL, and LDL in mice that are fed an HFD. The presence of important phenolic compounds in the walnut extract may be responsible for normalizing the serum lipid profile. In our study, the walnut extract contained a high amount of gallic acid (25.95%), which is likely the main factor in reducing hepatic lipids. Gallic acid also increased the activities of antioxidant enzymes and reduced lipid buildup in the liver of mice with HFD‐induced steatosis. This improvement in lipogenesis was achieved by downregulating the gene miR‐34a‐5p (Lee et al., 2022). Similarly, the findings Chao et al. (2021) also indicated that the hepatoprotective action of gallic acid in diabetic mice is partly mediated by blocking aberrant metabolic pathways involving glucose, amino acids, lipids, purines, and pyrimidines.

It has been observed that the decrease in SOD and CAT activities, along with an increase in MDA levels, is the result of oxidative imbalance, DNA damage, and enzyme damage. Previous reports have indicated the occurrence of oxidative stress in the tissues of HFD mice. According to Shoaib et al. (2023), prolonged oxidative stress causes lipid peroxidation and damages liver tissue. In our current study, we found that CAT and SOD activity were reduced in the HFD‐diseased control group. However, the administration of WE at different concentrations significantly improved CAT and SOD activity, thereby protecting the liver tissues. Previous studies have shown that an HFD can lead to the formation of ROS, which attacks the unsaturated bonds of fatty acids and activates lipid peroxidation, resulting in the production of TBARS (Vial et al., 2011). Rusu et al. (2020) showed that walnut kernel and septum extracts improved the antioxidant status of the liver. Similarly, in our study, TBARS levels in the livers of HFD‐fed mice significantly increased compared to the control group. However, this increase significantly declined in the groups treated with walnut extract, suggesting that WE treatment may reduce ROS generation and prevent hepatic damage.

Numerous studies have reported that the production of inflammatory cytokines, including TNF‐β, IL‐1β, and IL‐6, is what causes inflammation brought on by a high‐fat diet (Yao et al., 2016). In the present study, it was observed that HFD could significantly increase the expression of inflammatory cytokines, specifically IL‐6, and TNF‐α. However, in walnut extract, the presence of phenolic compounds led to the downregulation of these inflammatory markers. Gallic acid (Bai et al., 2021), quercetin, and catechin (Li et al., 2019) have been identified as anti‐inflammatory agents that act through the mitogen‐activated protein kinase (MAPK) and NF‐kB signaling pathways. The study of these pathways was the major limitation of the present study. However, it is believed that phenolic compounds in walnuts may have followed the same signaling pathways for the protection of the liver.

## 5 CONCLUSIONS

In conclusion, the HFD mice model was used to explore the potential anti‐inflammatory properties of the selected walnut extract in repairing HFD‐induced liver injury. This study also aimed to investigate the underlying molecular mechanisms through which the walnut extract inhibits inflammatory responses. Further research is necessary to fully understand the mechanism of action using genes of mitogen‐activated protein kinase (MAPK) and NF‐kB signaling pathways. The collective evidence presented in this study suggests that the walnut extract effectively reduces the expression of pro‐inflammatory markers, such as TNF‐α and IL‐6. Therefore, the walnut extract may play a unique role in promoting the recovery of liver damage caused by a high‐fat diet.

## AUTHOR CONTRIBUTIONS

**Gauhar Ali:** Data curation (equal); formal analysis (equal); investigation (equal); methodology (supporting); validation (equal); visualization (supporting); writing – original draft (equal). **Alam Zeb:** Conceptualization (lead); data curation (equal); formal analysis (equal); funding acquisition (lead); investigation (equal); methodology (lead); supervision (lead); writing – review and editing (equal). **Muhammad Usman:** Formal analysis (equal); investigation (equal). **Salim Al‐Babili:** Resources (equal); supervision (supporting); writing – review and editing (equal).

## FUNDING INFORMATION

There was no formal funding received for this work.

## CONFLICT OF INTEREST STATEMENT

The authors declare that the research was conducted in the absence of any commercial or financial relationships that could be construed as a potential conflict of interest.

## INSTITUTIONAL REVIEW BOARD STATEMENT

The study protocols were approved by the ethics committee of the Department of Biotechnology, following Helsinki standards. The research was subsequently accepted by the Graduate Studies Committee of the Department and the Advanced Studies and Research Board of the University of Malakand (No. UOM/Admin/2022/955).

## Supporting information


Data S1.

## Data Availability

The data presented in this study are available on request from the corresponding author.
