# Supplementary material for: Walnut extract protects against hepatic inflammation and toxicity induced by a high‐fat diet
Source: Food Sci Nutr. 2024 Sep 2;12(10):8340–52. doi: 10.1002/fsn3.4405 (PMC11521631; doi:10.1002/fsn3.4405)
Supplement: Supplementary file 1 — Data S1. [file FSN3-12-8340-s001.docx]

**Walnut extract protects against hepatic inflammation and toxicity induced by a high-fat diet**

**Gauhar Ali**^1^**, ^*^Alam Zeb**^2,3^**, Muhammad Usman**^4^, **Salim Al-Babili**^3^

**Affiliations**

1. Department of Biotechnology, University of Malakand, Chakdara, Pakistan ([gauharkhan41@yahoo.com](mailto:gauharkhan41@yahoo.com))
2. Bioactive Lab, Centre of Excellence for Sustainable Food Security, King Abdullah University of Science and Technology, Thuwal, Kingdom of Saudi Arabia (Salim.Babili@kaust.edu.sa)
3. Department of Biochemistry, University of Malakand, Chakdara, Pakistan ([azeb@uom.edu.pk](mailto:azeb@uom.edu.pk))
4. Department of Basic Sciences, University of Veterinary and Animals Sciences, Narowal, Pakistan ([Usman.asif@uvas.edu.pk](mailto:Usman.asif@uvas.edu.pk))

**Correspondence:**

*The Bioactive Lab, Centre of Excellence for Sustainable Food Security, King Abdullah University of Science and Technology, Thuwal 23955-6900, Kingdom of Saudi Arabia, Email: [alam.zeb@kaust.edu.sa](mailto:alam.zeb@kaust.edu.sa); azebuom@gmail.com

**Table S1**: List of the primers used in the study.

| Marker | Sequence | Reverse/Forward | Tm | GC% | Product length (bp) | Gene Accession Number |
| --- | --- | --- | --- | --- | --- | --- |
| TNF-α | 5’-ATGGGCTCCCTCTCATCAGT-3’ | Forward | 60.03 | 55.00 | 106 | NM_013693.3 |
|  | 5’-GCTTGGTGGTTTGCTACGAC-3’ | Reverse | 60.03 | 55.00 |  |  |
| IL-6 | 5’-CCCACCAGGAACGAAAGTCA-3’ | Forward | 59.89 | 55.00 | 70 | NM_001314054.1 |
|  | 5’-ACTGGCTGGAAGTCTCTTGC-3’ | Reverse | 59.96 | 55.00 |  |  |
| GAPDH | 5’-G ACTCCACTCACGGCAAATTC-3’ | Forward | 59.5 | 52.38 | 171 | NM_001411843.1 |
|  | 5’-TCTCCATGGTGGTGAAGACA-3’ | Reverse | 58.3 | 50.00 |  |  |

**Figure S1**: GC-MS chromatogram of tallow. Each peak with retention is a compound, as shown in Table S1.

**Table S2**: Composition of tallow studied using GC-MS.

| Peak | R.T (min) | Name | Total (%) |
| --- | --- | --- | --- |
| 1 | 2.653 | Ethylbenzene | 0.369 |
| 2 | 15.374 | Tetradecanoic acid | 1.581 |
| 3 | 16.106 | Pentadecanoic acid | 0.376 |
| 4 | 17.173 | E-9-Hexadecenoic acid | 1.723 |
| 5 | 17.468 | n-Hexadecanoic acid | 13.447 |
| 6 | 18.335 | Heptadecanoic acid | 0.757 |
| 7 | 18.669 | (E)-9-Octadecenoic acid | 0.337 |
| 8 | 19.108 | Oleic acid | 26.599 |
| 9 | 19.163 | NAE 15:2 | 7.653 |
| 10 | 19.337 | Octadecanoic acid | 13.238 |
| 11 | 19.392 | (Z,Z)-9,12-Octadecadienoic acid | 0.745 |
| 12 | 21.387 | Tetracosane | 0.193 |
| 13 | 22.283 | Bis(2-ethylhexyl) phthalate | 29.41 |
| 14 | 22.923 | Eicosane | 0.191 |
| 15 | 23.302 | (Z)-9-Octadecenoic acid | 0.25 |
| 16 | 28.329 | Cholesterol | 0.57 |
